# Is shock impedance value alone to be considered a good predictor for shock efficacy in subcutaneous implantable cardioverter defibrillator?

**DOI:** 10.1002/ccr3.1408

**Published:** 2018-02-07

**Authors:** Enrico Di Girolamo, Nanda Furia, Massimiliano Faustino, Marianna Appignani, Giampiero Arcari, Alessandro Angelini, Loris Spadoni

**Affiliations:** ^1^ Pacing and Electrophysiology Unit “Santissima Annunziata” Hospital Chieti Italy; ^2^ Boston Scientific Italy Milan Italy

**Keywords:** shock efficacy, shock impedance, subcutaneous defibrillator

## Abstract

Subcutaneous implantable cardioverter defibrillator (S‐ICD) is easy to implant, with poor risks of the patient. However, fat is a poor current conductor and increases defibrillation threshold. As shock impedance alone should not be considered a good efficacy predictor of an S‐ICD system, an X‐ray latero‐lateral view for lead position should be achieved.

## Introduction

Boston Scientific developed a new concept for implantable defibrillator, in which the defibrillation lead is implanted outside of the heart, under the skin. This lead leaves both the heart and the vessels untouched, thus to avoid possible lead‐related failure or complication 1. The can is implanted on the left side, along the midauxiliary line, in three different locations: subcutaneous, intermuscolar (between serratus anterior and latissimus dorsi), or undermuscolar (under serratus anterior, over the ribs) 2. The lead is tunneled from this side to parasternal left or right position (depending on heart position and ECG screening results) 3.

After the implant procedure, in order to confirm the optimal placement, the system efficacy is tested by induction of ventricular fibrillation and treatment by a 65 J (or less) biphasic shock, delivered by the subcutaneous defibrillator itself. Several studies have reported high rates of cardioversion efficacy during acute test (96–98.2%) 4, 5, 6 After defibrillation threshold test, physicians can obtain shock impedance value in addition to shock efficacy evaluation only. Usually, this value ranges between 50 and 80 Ohms, and it is correlated with an optimal system placement. As a matter of fact, a low shock impedance value suggests the absence or poor fat under the coil lead and the can.

Recently, in order to avoid ventricular defibrillation induction, some physicians prefer to deliver a manual synchronous 10 J shock to evaluate the shock impedance. If this value is <100 Ohms, the implant is considered to have been placed correctly.

We want to describe this case report as, despite low shock impedance, a 65 J shock (delivered in both standard and reverse configuration) was not able to convert the induced ventricular fibrillation.

## Case Report

A 28‐year‐old male patient was admitted in our hospital after a syncopal ventricular arrhythmia that requested external DC shock in the emergency room to restore sinus rhythm and hemodynamics.

In sinus rhythm, the patient showed a type 1 Brugada pattern at rest ECG.

According to his young age and no need for pacing, we suggested an subcutaneous implantable cardioverter defibrillator (S‐ICD) system implantation.

In June 2017, the S‐ICD system (EMBLEM A219) was implanted creating an intermuscular pocket for the device, with the lead positioned vertically in the subcutaneous tissue, 2 cm sternal midline. left. We preferred to use a two incision technique in order to avoid the superior scar in this young patient. The system position was previously defined by positioning a demo over the patient's chest, in order to check by fluoroscopy the best position for can and lead, thus establishing landmarkers.

**Figure 1:**
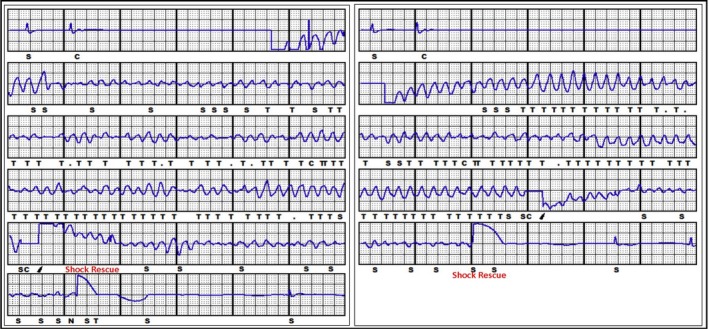
First and second failed induction test.

After incisions closure, the S‐ICD selected the secondary sensing vector as the best vector, according to the evaluation of signals only in supine position, immediately prior to the induction test. This vector was permanently programmed with an 1x gain, and the S‐ECG template was acquired. The induction test was performed inducing ventricular fibrillation through the device itself, but the S‐ICD was unable to restore sinus rhythm by a 65 J biphasic shock, in standard polarity, despite 69 Ohms shock impedance, and a 200 J shock by external defibrillator was required. A new attempt was performed programming the first shock modality in reverse polarity. Again, after induction, the S‐ICD was unable to restore sinus rhythm by 65 J and a new 200 J external shock was required (the subcutaneous shock impedance was still 69 Ohms) (Fig. 1).

**Figure 2:**
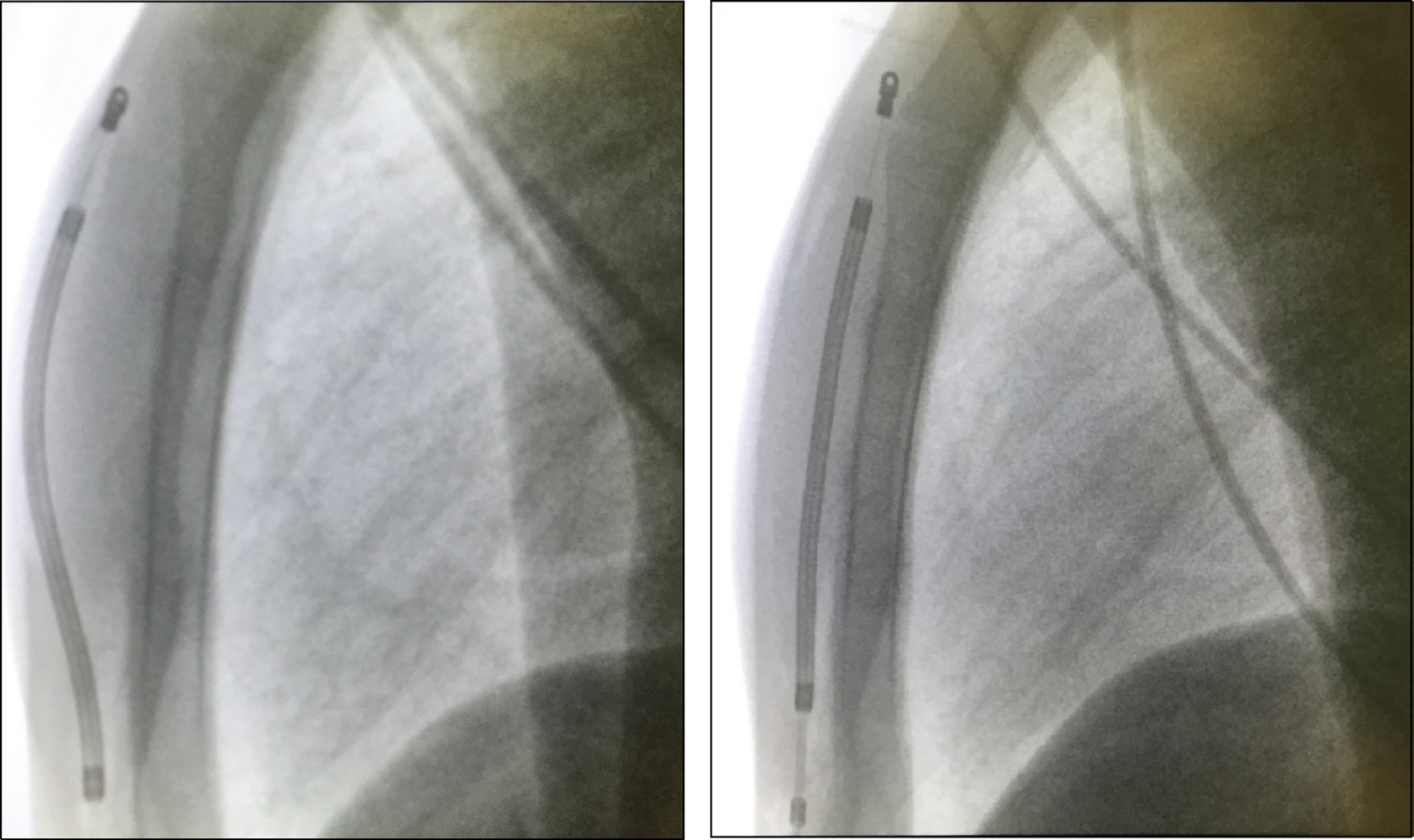
Lead position before and after replacement in LL view.

At this point, we decided to reassess the system position by fluoroscopy. In posteroanterior projection, the system seemed in the same position with respect to the probe. Looking in latero‐lateral projection, we noted that not all the coil lead was on the fascial plane (Fig. 2).

**Figure 3:**
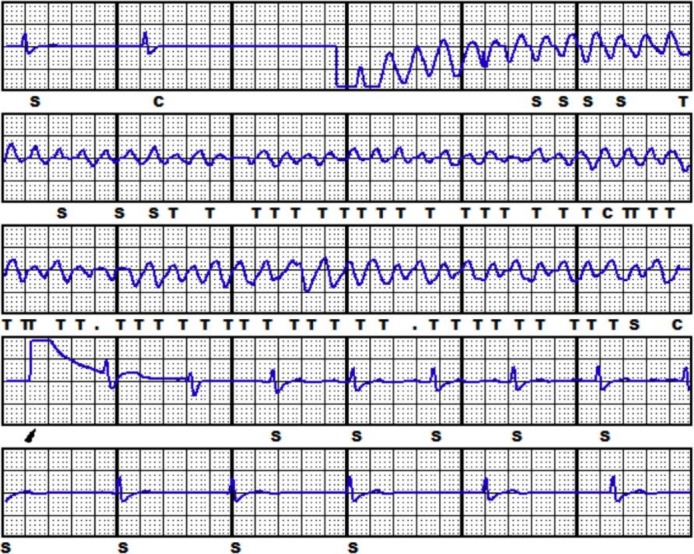
Third (final) induction test.

Probably, during vertical tunnelization, the tunneling tool was moved up, thus positioning the tip of the lead into the fat after under the skin. As showed by mathematical simulation 7, the fat is a poor current conductor and its presence between coil and sternal facial plane decreases considerably the defibrillation efficacy. Thus, a lead repositioning, keeping the tunneling tool down for all the way, using fingers on the surface of the sternum over the tip of the tunneling tool to help guide the tip and stay close to the facial plane, was performed. The new lead position was checked by fluoroscopy in latero‐lateral projection (Fig. 2 *)* and, after xyphoide incision closure, a new induction test was performed. The arrhythmia was induced by 2 sec alternate current delivery through the device. The S‐ICD promptly restored sinus rhythm by a single 65 J shock in standard polarity (Fig. 3). The shock impedance value decreased to 44 Ohms.

## Discussion

The current generation of S‐ICD was intentionally designed to avoid long‐term lead complication, using a defibrillation lead placed under the skin, which leaves both the heart and the vessel completely untouched. This system is easy and safe to implant and easier to remove with poor risks of the patient.

As showed by mathematical simulation and according to the manufacturer's instructions 8, the can and the lead must be placed in contact with the facial planes. The fat between can and chest or between coil lead and chest reduces shock efficacy, increasing defibrillation threshold.

After implantation, before performing induction test or in case of shock failure, we suggest to check the lead position by fluoroscopy in latero‐lateral projection, in order to insure fat absence under the system.

If induction test is not performed, we suggest to deliver a 10 J (or more) shock in synchronous manner by S‐ICD (as a cardioversion) just to evaluate shock impedance. Shock impedance value alone should not be considered a safe predictor for shock efficacy as the absence of fat tissue under the coil lead has not been evaluated by fluoroscopy.

## Conflict of Interest

Giampiero Arcari, Alessandro Angelini and Loris Spadoni are employees of Boston Scientific. No other conflicts of interests exist.

## Authorship

EDG: was the first surgeon who performed the S‐ICD implantation. NF: was the second surgeon who performed the S‐ICD implantation. MF: was the third surgeon who performed the lead replacement together with the first surgeon. MA: was the I.C.U. Cardiologist who admitted, followed, and proposed the patient for the S‐ICD, GA and AA: was the technical supply during implantation and lead repositioning. LS: was the technical and bibliographic support for paper editing.
